# Functional Characterization of the Lactolisterin BU Gene Cluster of *Lactococcus lactis* subsp. *lactis* BGBU1-4

**DOI:** 10.3389/fmicb.2018.02774

**Published:** 2018-11-15

**Authors:** Marija Miljkovic, Jelena Lozo, Nemanja Mirkovic, Paula M. O’Connor, Milka Malesevic, Branko Jovcic, Paul D. Cotter, Milan Kojic

**Affiliations:** ^1^Laboratory for Molecular Microbiology, Institute of Molecular Genetics and Genetic Engineering, University of Belgrade, Belgrade, Serbia; ^2^Faculty of Biology, University of Belgrade, Belgrade, Serbia; ^3^Teagasc Food Research Centre, Moorepark, Fermoy, Ireland; ^4^APC Microbiome Ireland, University College Cork, Cork, Ireland

**Keywords:** bacteriocin, lactolisterin BU operon, ABC transporter, limited immunity, translational coupling

## Abstract

The gene cluster responsible for the production of the aureocin A53-like bacteriocin, lactolisterin BU, is located on plasmid pBU6 in *Lactococcus lactis* subsp. *lactis* BGBU1-4. Heterologous expression of pBU6 confirmed that production and limited immunity to lactolisterin BU were provided by the plasmid. Comparative analysis of aureocin A53-like operons revealed that the structural genes shared a low level of identity, while other genes were without homology, indicating a different origin. Subcloning and expression of genes located downstream of the structural gene, *lliBU*, revealed that the lactolisterin BU cluster consists of four genes: the structural gene *lliBU*, the *abcT* gene encoding an ABC transporter, the *accL* gene encoding an accessory protein and the *immL* gene which provides limited immunity to lactolisterin BU. Reverse transcription analysis revealed that all genes were transcribed as one polycistronic mRNA. Attempts to split the lactolisterin BU operon, even when both parts were under control of the P*lliBU* promoter, were unsuccessful indicating that expression of lactolisterin BU is probably precisely regulated at the translational level by translational coupling and is possible only when all genes of the operon are *in cis* constellation. Two ρ-independent transcription terminators were detected in the lactolisterin BU operon: the first in the intergenic region of the *lliBU* and *abcT* genes and the second at the end of operon. Deletion of the second transcription terminator did not influence production of the bacteriocin in lactococci.

## Introduction

Ribosomal synthesis of antimicrobial peptides or peptide complexes, known as bacteriocins, presents a successful strategy for the reduction of microbial competitors to obtain more nutrients and living space in the environment (Cotter et al., 2013). They can be active against closely related bacterial species or different bacterial genera, and the producer protects itself by synthesis of specific immunity proteins (Kjos et al., 2014). Bacteriocins produced by lactic acid bacteria (LAB) are generally regarded as natural and suitable for consumption as they have been present in many foods and beverages since ancient times (Cleveland et al., 2001). Numerous studies reported that bacteriocins may be applied in the food industry to extend food preservation time (Cotter et al., 2005; Yang et al., 2014; Ahmad et al., 2017). A number of studies consider potential clinical applications of bacteriocins for maintenance of human health as a part of anti-infection or even are able to cause apoptotic and necrotic death of cancer cells *in vitro* (Lagos et al., 2009; Yang et al., 2014; Ahmad et al., 2017; Baindara et al., 2017). In addition to being administered as food supplements, some bacteriocins may be produced at the site of infection by probiotic bacteria (Gotteland et al., 2008).

Bacteriocins differ in molecular mass, structure, biochemical and genetic characteristics, modes of action and target cell receptors. Based on these criteria, bacteriocins from LAB are generally classified into two main classes: Class I bacteriocins (lantibiotics) contain unusual amino acids such as lanthionine and dehydrated amino acids as a result of post-translational modifications and Class II bacteriocins consisting of unmodified or peptides with minor modifications. Furthermore, Class II bacteriocins are subdivided into four subclasses: pediocin-like bacteriocins (class IIa), two-peptide bacteriocins (class IIb), cyclic bacteriocins (IIc), and linear non-pediocin-like bacteriocins (class IId) (Cotter et al., 2013). Recently a new modified classification of LAB bacteriocins was proposed (Alvarez-Sieiro et al., 2016) with two main differences: (a) the introduction of class III which includes thermo-labile bacteriolysins and non-lytic bacteriocins with a molecular mass greater than 10 kDa and (b) leaderless bacteriocins are classified as subclass IIc. Class II bacteriocins, in general, are small (37–58 amino acids) cationic peptides with pIs varying from 8 to 11. They contain a relatively high number of small amino acids such as glycine and alanine, giving them a high degree of conformational freedom (Kaiser and Montville, 1996). Most bacteriocins are synthesized as a pro-peptide with an N-terminal leader sequence, which is cleaved by a dedicated ABC transporter or the Sec system during export from cells. However, there is a group of so-called leaderless bacteriocins, which are synthesized without an N-terminal leader sequence and the mechanism behind their export is still unknown. Leaderless bacteriocins are often relatively small (30–50 amino acids) and without modifications (Ovchinnikov et al., 2017).

Bacteriocin genetic systems can be located on chromosomes, plasmids or conjugative transposons (Engelke et al., 1992; Rauch and De Vos, 1992; De Vos et al., 1995; Kojic et al., 2005). Class II bacteriocins are organized in operons consisting of at least four genes: a structural gene, an immunity gene, a transporter gene and an accessory protein gene (Nes et al., 1996; Mesa-Pereira et al., 2017) or in gene clusters. The structural bacteriocin gene encodes a pre-form of the bacteriocin containing an N-terminal leader sequence (except leaderless bacteriocins). Each bacteriocin has its own protein conferring immunity encoded by an immunity gene located next to or close downstream of the bacteriocin structural gene. The ABC transporter protein is responsible for transport of the bacteriocin from the cytoplasm to the outside of the membrane and for extracellular activation by cleavage of the leader sequence. An additional gene encoding an accessory protein (approximately 470 amino acids) is usually located next to the ABC transporter and is apparently required for the ABC-transporter-dependent translocation process (Nes et al., 1996). Expression of bacteriocin genes is usually regulated by external induction factors, in most cases small peptides secreted by the producer strain itself or by its competitors for the ecological resources. The production of class II bacteriocins is transcriptionally regulated by a three component quorum sensing system, which consists of an induction factor, a membrane-associate protein histidine kinase, and response regulators (Diep et al., 2003). Recently, it has been described for the first time that the expression of LsbB, a leaderless bacteriocin, is regulated by a transcription terminator sequence located downstream of the structural gene (Uzelac et al., 2015). However, in some cases, bacteriocin production depends on environmental conditions such as temperature, pH and free peptide concentration.

In this study, we investigated the function of the hypothetical genes within the lactolisterin BU gene cluster of *Lactococcus lactis* subsp. *lactis* BGBU1-4. Previously, it was shown that strain BGBU1-4 produces a novel, leaderless bacteriocin, lactolisterin BU, with strong antimicrobial activity against many species of Gram-positive bacteria, including important food spoilage and food-borne pathogens such as *Listeria monocytogenes*, *Staphylococcus aureus*, *Bacillus* sp. and streptococci. The production of lactolisterin BU is related to the smallest plasmid pBU6 of 6.2 kb, which contains nine open reading frames (ORFs): *repB*, *lliBU*, *abcT*, *hyp1*, *hyp2*, *hyp3*, *mobC*, *relM*, and *rnaY* (Lozo et al., 2017). An identity (BLAST) search within the lactolisterin BU operon recognized the product of the *abcT* gene as a sugar transporter, while other genes were designated as hypothetical. The aim of this work was to determine the function of the hypothetical genes (*hyp1*, *hyp2*, and *hyp3*) and their involvement in bacteriocin activity.

## Materials and Methods

### Bacterial Strains and Growth Conditions

The strains, their derivatives and plasmids used in this study are listed in Table 1. *Lactococcus lactis* strains were grown in M17 medium (Merck GmbH, Darmstadt, Germany) supplemented with 0.5% glucose (GM17) at 30°C. *Escherichia coli* strains (DH5α and EC101) were grown in Luria Bertani broth (LB) at 37°C with aeration. To each medium, agar (1.5%; Torlak, Belgrade, Serbia) was added for use as a solid medium. The following antibiotic concentrations were used: ampicillin, 100 μg/ml (*E. coli*); erythromycin, 10 μg/ml (lactococci) and 300 μg/ml (*E. coli*); chloramphenicol, 10 μg/ml (lactococci) and 35 μg/ml (*E. coli*). The 5-bromo-4-chloro-3-indolyl-β-D-galacto-pyranoside (X-Gal) (Fermentas, Vilnius, Lithuania) was added to LB medium plates for blue/white color screening of colonies with cloned fragments at a final concentration of 80 μg/ml (lactococci) and 40 μg/ml (*E. coli*). Ortho-nitrophenyl-β-galactoside (ONPG) was used for the β-galactosidase assay (Sigma Chemical Co., St. Louis, MO, United States).

**Table 1:** Bacterial strains and plasmids used in this study.

Strains, plasmids, and derivatives	Relevant characteristics	Source
***Lactococcus lactis* subsp. *lactis***		
BGBU1-4	Bacteriocin (lactolisterin BU) producer	Golić et al., 2013
BGMN1-596	Plasmid free derivative of *L. lactis* subsp. *lactis* BGMN1-5	Gajic et al., 1999
***Lactococcus lactis* subsp. *cremoris***		
MG7284	Prt^−^, Lac^−^, Bac^r^, Fus^r^, Spc^r^	Gasson, 1983
***Escherichia coli***		
DH5α	*supE44 ΔlacU169* (*ø80 lacZΔM15*) *hsdR17 recA1 endA1 gyrA96 thi-1 relA1*	Hanahan, 1983
EC101	JM101 containing *repA* gene of pWV01 in chromosome	Law et al., 1995
**Plasmids**		
pBU6	6.2 kb, natural plasmid carrying lactolisterin BU gene cluster from strain BGBU1-4	Lozo et al., 2017
pBluescriptT/A	2994 bp, Amp^r^, PCR cloning vector	Uzelac et al., 2015
pAZIL	7,109 bp, Em^r^, shuttle cloning vector	Kojic et al., 2011
pNZ8150	Cm^r^, NICE expression vector	Mierau and Kleerebezem, 2005
pNZ8150-lacZ1	pNZ8150 carrying *lacZ1* gene	Vukotic et al., 2015
pAZIL-SP	*Sac*I/*Pvu*II fragment (*lliBU*, *abcT*, *hyp1*, *hyp2*, *hyp3* and part of *mobC*) from pBU6 into pAZIL predigested with *Sac*I/*Sma*I	This work
pAZIL-SB	*Sac*I/*Bgl*II fragment (*lliBU*, *abcT*, *hyp1* and *hyp2*) from pBU6 into pAZIL predigested with *Sac*I/*Bam*HI	This work
pAZIL-SS	*Sac*I/*Sca*I fragment (*lliBU* and *abcT*) from pBU6 into pAZIL predigested with *Sac*I/*Sma*I	This work
pAZIL-pBU6P-1	pBU6 digested with *Pvu*II into pAZIL predigested with *Sma*I (first orientation; under P*lacZ*)	This work
pAZIL-pBU6P-5	pBU6 digested with *Pvu*II into pAZIL predigested with *Sma*I (second orientation)	This work
pAZIL-pBU6B-2	pBU6 digested with *Bgl*II into pAZIL predigested with *Bam*HI (first orientation; under P*lacZ*)	This work
pAZIL-pBU6B-4	pBU6 digested with *Bgl*II into pAZIL predigested with *Bam*HI (second orientation)	This work
pBluescriptT/A-PlliBU	PCR-amplified fragment carrying P*lliBU* cloned into pBluescriptT/A ^∗^	This work
pNZ8150-h1	*hyp1* cloned into pNZ8150 under P*lliBU* ^∗^	This work
pNZ8150-h2	*hyp2* cloned into pNZ8150 under P*lliBU* ^∗^	This work
pNZ8150-h1+h2	*hyp1 and hyp2* cloned into pNZ8150 under P*lliBU* ^∗^	This work
pNZ8150-h3	*hyp3* cloned into pNZ8150 under P*hyp3*^∗^	This work
pNZ8150-h3+mobC+relM+rnaY	*hyp3, mobC, relM* and *rnaY* cloned into pNZ8150 under P*hyp3* ^∗^	This work
pAZIL-EEB	pAZIL-SB partially digested with *Eco*RI (without fragment of 382 bp which containing P*lliBU* and part of the *lliBU* gene)	This work
pNZ8150-EEB	*Xba*I fragment from pAZIL-EEB carrying whole lactolisterin BU cluster with non-functional *lliBU* gene	This work
pAZIL-S-h1	PCR-amplified fragment carrying P*lliBU*, *lliBU*, *abcT* and *hyp1* cloned into pAZIL ^∗^	This work
pAZIL-S-h1+h2-TT2	PCR-amplified fragment carrying P*lliBU*, *lliBU*, *abcT*, *hyp1* and *hyp2* without TT2 cloned into pAZIL ^∗^	This work
pAZIL-S-h1+h2+1/2TT2	PCR-amplified fragment carrying P*lliBU*, *lliBU*, *abcT*, *hyp1* and *hyp2* with ½ TT2 cloned into pAZIL ^∗^	This work
pAZIL-S-h1+h2+TT2	PCR-amplified fragment carrying P*lliBU*, *lliBU*, *abcT*, *hyp1* and *hyp2* with TT2 cloned into pAZIL ^∗^	This work
pNZ8150-PlliBU-lacZ1	P*lliBU* cloned into pNZ8150-lacZ1 ^∗^	This work
pNZ8150-PlcnB-lacZ1	P*lcnB* cloned into pNZ8150-lacZ1 ^∗^	This work
**Derivatives**		
MG7284/pBU6	MG7284 transformed with plasmid pBU6 (producer of lactolisterin BU)	Lozo et al., 2017
MG7284/pAZIL-SP	MG7284 transformed with pAZIL-SP	This work
MG7284/pAZIL-SB	MG7284 transformed with pAZIL-SB	This work
MG7284/pAZIL-SS	MG7284 transformed with pAZIL-SS	This work
MG7284/pAZIL-pBU6P-1	MG7284 transformed with pAZIL-pBU6P-1	This work
MG7284/pAZIL-pBU6P-5	MG7284 transformed with pAZIL-pBU6P-5	This work
MG7284/pAZIL-pBU6B-2	MG7284 transformed with pAZIL-pBU6B-2	This work
MG7284/pAZIL-pBU6B-4	MG7284 transformed with pAZIL-pBU6B-4	This work
MG7284/pNZ8150-h1	MG7284 transformed with pNZ8150-h1	This work
MG7284/pAZIL-SS+pNZ8150-h1	MG7284/pAZIL-SS transformed with pNZ8150-h1	This work
MG7284/pNZ8150-h2	MG7284 transformed with pNZ8150-h2	This work
MG7284/pAZIL-SS+pNZ8150-h2	MG7284/pAZIL-SS transformed with pNZ8150-h2	This work
MG7284/pNZ8150-h1+h2	MG7284 transformed with pNZ8150-h1+h2	This work
MG7284/pAZIL-SS+pNZ8150-h1+h2	MG7284/pAZIL-SS transformed with pNZ8150-h1+h2	This work
MG7284/pNZ8150-h3	MG7284 transformed with pNZ8150-h3	This work
MG7284/pAZIL-SS+pNZ8150-h3	MG7284/pAZIL-SS transformed with pNZ8150-h3	This work
MG7284/pAZIL-SB+pNZ8150-h3	MG7284/pAZIL-SB transformed with pNZ8150-h3	This work
MG7284/pAZIL-SP+pNZ8150-h3	MG7284/pAZIL-SP transformed with pNZ8150-h3	This work
MG7284/pNZ8150-h3+mobC+relM+rnaY	MG7284 transformed with pNZ8150-h3+mobC+relM+rnaY	This work
MG7284/pAZIL-SS+pNZ8150-h3+mobC+relM+rnaY	MG7284/pAZIL-SS transformed with pNZ8150-h3+mobC+relM+rnaY	This work
MG7284/pAZIL-SB+pNZ8150-h3+mobC+relM+rnaY	MG7284/pAZIL-SB transformed with pNZ8150-h3+mobC+relM+rnaY	This work
MG7284/pNZ8150-EEB	MG7284 transformed with pNZ8150-EEB	This work
MG7284/pAZIL-SS+ pNZ8150-EEB	MG7284/pAZIL-SS transformed with pNZ8150-EEB	This work
MG7284/pAZIL-S-h1+h2-TT2	MG7284 transformed with pAZIL-S-h1+h2-TT2	This work
MG7284/pAZIL-S-h1+h2+1/2TT2	MG7284 transformed with pAZIL-S-h1+h2+1/2TT2	This work
MG7284/pAZIL-S-h1+h2+TT2	MG7284 transformed with pAZIL-S-h1+h2+TT2	This work
MG7284/pNZ8150-lacZ1	MG7284 transformed with pNZ8150-lacZ1	Vukotic et al., 2015
MG7284/pNZ8150-PlliBU-lacZ1	MG7284 transformed with pNZ8150-PlliBU-lacZ1	This work
MG7284/pNZ8150-PlcnB-lacZ1	MG7284 transformed with pNZ8150-PlcnB-lacZ1	This work

*Amp^r^, resistance to ampicillin; Bac^r^, bacteriocin resistant; Cm^r^, resistance to chloramphenicol; Em^r^, resistance to erythromycin; Fus^r^, fusaric acid resistant; Lac^−^, lactose utilization negative; Prt^−^, proteinase negative; Spc^r^, resistance to spectinomycin; ^∗^ for details, see the Section “Construction of Derivatives.”*

All strains carrying constructs were stored in growth medium containing 15% glycerol (Sigma Chemie GmbH, Deisenhofen, Germany) at −80°C.

### DNA Manipulations

For plasmid isolation from *E. coli* a GeneJET Plasmid Miniprep kit was used according to the manufacturer’s recommendations (Thermo Fisher Scientific, Waltham, MA, United States). Plasmids from lactococci were isolated by a modification of the method described by O’Sullivan and Klaenhammer (1993). Digestion with restriction enzymes was conducted according to the supplier’s instructions (Thermo Fisher Scientific, Waltham, MA, United States). The DNA fragments from agarose gels were purified using QIAqick Gel extraction kit as described by the manufacturer (Qiagen, Hilden, Germany). DNA was ligated with T4 DNA ligase according to the manufacturer’s recommendations (Agilent Technologies, United States).

Platinum^TM^ *Taq* DNA Polymerase High Fidelity (Thermo Fisher Scientific, Waltham, MA, United States) was used to amplify DNA fragments using a GeneAmp PCR System 2700 thermal cycler (Applied Biosystems, Foster City, CA, United States). The PCR programs consisted of initial denaturation (5 min at 96°C), 30 cycles of denaturation (30 s at 96°C), annealing (30 s at the appropriate temperature (Table 2) and polymerization (2 min, 1 min or 30 s at 68°C; depending on the length of the amplified fragment), and an additional extension step of 5 min at 68°C. Sets of specific primers used in this study are listed in Table 2. PCR products were purified with a Thermo Scientific PCR Purification Kit according to the manufacturer’s protocol (Thermo Fisher Scientific, Waltham, MA, United States). For cloning PCR products a pBluescriptT/A (Uzelac et al., 2015) or pAZIL vector were used.

**Table 2:** Primers used in this study.

Primer name	Sequence (5′ to 3′)	Product size (bp)	Temperature of annealing	Reference
PBU1-4-Fw	GGACTAAAAACAAGTAAC	136	42°C	This work
PBU1-4-Rev	CATATGTAAGAGTACCTCC			
lliBU-Fw	ATGTGGGGTAGAATTCTTGG	134	51°C	This work
lliBU-Rev	CATTCCAAGCTTATATTTTAACCCC			
lliBU-Fw	ATGTGGGGTAGAATTCTTGG	830	48°C	This work
abcT-Rev	CAAATAATTCACGCTCC			
lliBU-Fw	ATGTGGGGTAGAATTCTTGG	1,486	44°C	This work
hyp1-Rev	GAGTATCTACTTTTCAAG			
lliBU-Fw	ATGTGGGGTAGAATTCTTGG	2,004	54°C	This work
middle_the_loop_hyp2-Rev	TGGCGGAAATATGTCCC			
abcT-Fw	CACAATTACCCAGAATTC	663	44°C	This work
abcT-Rev	CAAATAATTCACGCTCC			
abcT-Fw	CACAATTACCCAGAATTC	1,319	44°C	This work
hyp1-Rev	GAGTATCTACTTTTCAAG			
abcT-Fw	CACAATTACCCAGAATTC	1,837	48°C	This work
middle_the_loop_hyp2-Rev	TGGCGGAAATATGTCCC			
hyp1PstI-Fw	CTGCAGTATTGGAGCGTGAATTATTTG	677	53°C	This work
hyp1XbaI-Rev	TCTAGATTTCAAGTCCTATACCTTCG			
hyp2PstI-Fw	CTGCAGGTTAGAAACGAAGGTATAGG	570	55°C	This work
hyp2XbaI-Rev	TCTAGATTTGGCGGAAATATGTCC			
hyp3BglII-Fw	GGAGATCTACAAAACTTAAGG	615	48°C	This work
hyp3-Rev	GTAAGAATCCTCCTCTCG			
hyp3BglII-Fw	GGAGATCTACAAAACTTAAGG	2,585	48°C	This work
ribonucleaseY-Rev	GGCTCACTCAGTTCGGTCAGGG			
SacI_before_prom-Fw	GAGTGGTTAGGAGCTCC	1,860	41°C	This work
hyp1-Rev	GAGTATCTACTTTTCAAG			
SacI_before_prom-Fw	GAGTGGTTAGGAGCTCC	2,366	44°C	This work
before_the_loop- hyp2-Rev	GTCCCAATTAATACTAGC			
SacI_before_prom-Fw	GAGTGGTTAGGAGCTCC	2,378	51°C	This work
middle_the_loop_hyp2-Rev	TGGCGGAAATATGTCCC			
SacI_before_prom-Fw	GAGTGGTTAGGAGCTCC	2,434	52°C	This work
after_the_loop- hyp2-Rev	CGCTTCCCCGAACCCCCG			
LcnB-Fw	AGTTATTAACATTTGTTAACG	77	38°C	This work
LcnB-Rev	CATAATAATCTCCTTATTTTTATAAATC			

*Introduced restriction sites are indicated by underlined sequences.*

#### Construction of Derivatives

**FIGURE 1:**
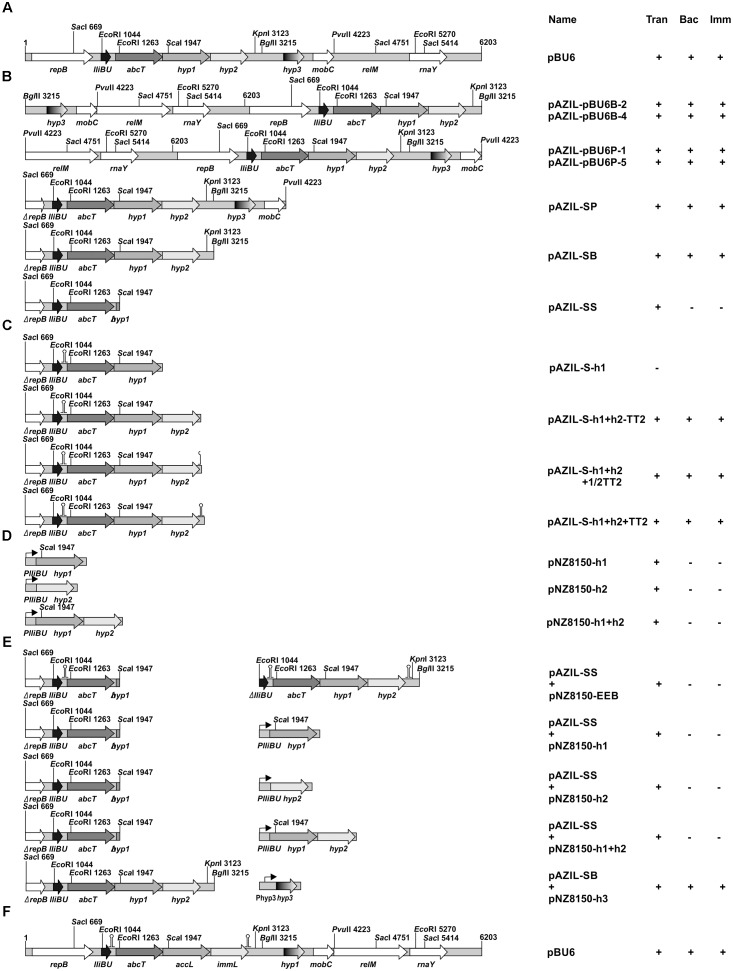
Schematic representation of the construction of appropriate constructs. **(A,F)** Linear gene map of the pBU6 plasmid carrying the lactolisterin BU cluster; **(B–E)** the scheme of construction of clones used for analysis of influence of different genes/regions on the expression of the *lliBU* gene. Relevant restriction sites are indicated. The size and orientation of predicted ORFs are indicated by arrows. +/−, presence/absence of Trans (transformants); Bac, bacteriocin activity; Imm, expressed limited immunity.

The *Sac*I/*Pvu*II fragment from pBU6, containing the lactolisterin BU operon (*lliBU*, *abcT*, *hyp1* and *hyp2*), *hyp3* and part of *mobC* was cloned into pAZIL vector predigested with *Sac*I/*Sma*I restriction enzymes giving construct pAZIL-SP (Figure 1B and Table 1). The fragment *Sac*I/*Bgl*II from pBU6, containing just the lactolisterin BU operon (*lliBU*, *abcT*, *hyp1* and *hyp2*) was cloned into pAZIL vector predigested with *Sac*I/*Bam*HI restriction enzymes giving construct pAZIL-SB (Figure 1B and Table 1). The *Sac*I/*Sca*I fragment from pBU6, containing *lliBU* and *abcT* was cloned into pAZIL vector predigested with *Sac*I/*Sma*I restriction enzymes giving construct pAZIL-SS (Figure 1B and Table 1). In addition, (*i*) *Pvu*II fragment from pBU6 (containing whole pBU6 plasmid; *Pvu*II restriction site cut of the start of *mobC*) was cloned into pAZIL vector predigested with *Sma*I restriction enzyme. Obtained constructs were named pAZIL-pBU6P-1 and pAZIL-pBU6P-5 (both possible orientations) (Figure 1B and Table 1); (*ii*) *Bgl*II fragment from pBU6 (containing whole pBU6 plasmid; *Bgl*II restriction site is within the intergenic region of the *hyp2* and *hyp3* genes) was cloned into pAZIL vector predigested with *Bam*HI restriction enzyme. Obtained constructs, were named pAZIL-pBU6B-2 and pAZIL-pBU6B-4 (both possible orientations) (Figure 1B and Table 1). MG7284 was transformed with each of the constructs and the obtained derivatives were used as bacteriocin producers in the agar well diffusion assay.

The lactolisterin BU promoter, P*lliBU*, was amplified by PCR using primers PBU1-4-Fw and PBU1-4-Rev where pBU6 was used as a template (Table 2). The obtained fragment was cloned into pBluescriptT/A vector giving a construct named pBluescriptT/A-PlliBU (Table 1). The *hyp1* gene was amplified from pBU6 using the hyp1PstI-Fw and hyp1XbaI-Rev primers (Table 2) and cloned into pBluescriptT/A vector (giving the construct pBluescriptT/A-h1). The *Xba*I fragment was transferred, downstream of the P*lliBU* promoter, into the pBluescriptT/A-PlliBU predigested with *Xba*I restriction enzyme, giving the construct pBluescriptT/A-PlliBU-h1. The *Sac*I/*Eco*RV fragment from pBluescriptT/A-PlliBU-h1 was transferred into pNZ8150 vector predigested with *Sac*I/*Sca*I restriction enzymes giving the construct pNZ8150-h1 (Figure 1D and Table 1). The *hyp2* gene was amplified from pBU6 plasmid using the hyp2PstI-Fw and hyp2XbaI-Rev primers (Table 2) and cloned into pBluescriptT/A vector giving the construct pBluescriptT/A-h2. The *Xba*I fragment was transferred, downstream of the P*lliBU* promoter, into the pBluescriptT/A-PlliBU predigested with *Xba*I restriction enzyme, giving the construct pBluescriptT/A-PlliBU-h2. The *Sac*I/*Eco*RV fragment from pBluescriptT/A-PlliBU-h2 was transferred into pNZ8150 vector predigested with *Sac*I/*Sca*I restriction enzymes giving the construct pNZ8150-h2 (Figure 1D and Table 1). A fragment containing the *hyp1* and *hyp2* genes was amplified from pBU6 plasmid using the hyp1PstI-Fw and hyp2XbaI-Rev primers (Table 2) and cloned into pBluescriptT/A vector giving the construct pBluescriptT/A-h1+h2. The *Xba*I fragment was transferred, downstream of the P*lliBU* promoter, into the pBluescriptT/A-PlliBU predigested with *Xba*I restriction enzyme, giving the construct pBluescriptT/A-PlliBU-h1+h2. The *Sac*I/*Eco*RV fragment from pBluescriptT/A-PlliBU-h1+h2 was transferred into pNZ8150 vector predigested with *Sac*I/*Sca*I restriction enzymes giving the construct pNZ8150-h1+h2 (Figure 1D and Table 1). The *hyp3* gene (with its promoter – P*hyp3*) was amplified by PCR using primers hyp3BglII-Fw and hyp3-Rev where pBU6 was used as a template (Table 2) and cloned into pBluescriptT/A vector, giving the construct pBluescriptT/A-h3. The fragment from pBluescriptT/A-h3 was transferred into pNZ8150 as *Pst*I/*Xba*I giving the construct pNZ8150-h3 (Table 1). Fragment containing the *hyp3*, *mobC*, *relM* and *rnaY* genes was amplified by PCR using primers hyp3BglII-Fw and rnaY-Rev where pBU6 was used as a template (Table 2) and cloned into pBluescriptT/A vector, giving the construct pBluescriptT/A-h3mobCrelMrnaY. The fragment from pBluescriptT/A-h3mobCrelMrnaY was transferred into pNZ8150 as *Pst*I/*Xba*I fragment giving the construct pNZ8150-h3+mobC+relM+rnaY (Table 1). MG7284 and MG7284/pAZIL-SS were transformed with each of the constructs (pNZ8150-h1, pNZ8150-h2, pNZ8150-h1+h2, pNZ8150-h3 and pNZ8150-h3+mobC+relM+rnaY) and the obtained derivatives (listed in Table 1) were used as bacteriocin producers in the agar well diffusion assay. MG7284 and MG7284/pAZIL-SB were transformed with the following constructs (pNZ8150-h3 and pNZ8150-h3+mobC+relM+rnaY) and the obtained derivatives (listed in Table 1) were used as bacteriocin producers in the agar well diffusion assay. MG7284/pAZIL-SP was transformed with pNZ8150-h3 and the obtained derivative (Table 1) was used for further experiments.

The construct pAZIL-SB was partially digested with *Eco*RI to remove a fragment of 382 bp (without P*lliBU* and first part of the *lliBU* gene) giving the construct pAZIL-EEB. In order to obtain pNZ8150-EEB, *Xba*I fragment from pAZIL-EEB (carrying all lactolisterin BU operon with non-functional *lliBU* gene) was recloned into pNZ8150 vector. MG7284 and MG7284/pAZIL-SS were transformed with pNZ8150-EEB and the obtained derivatives (Table 1) were used for further experiments.

A section of the operon from the *Sac*I restriction site (upstream of P*lliBU*) to the end of *hyp1* gene was amplified by PCR using primers SacI_before_prom-Fw and hyp1-Rev where pBU6 was used as a template (Table 2) and cloned into pAZIL vector (predigested with *Sma*I restriction enzyme), giving the construct pAZIL-S-h1 (Figure 1C and Table 1). The constructs pAZIL-S-h1+h2-TT2, pAZIL-S-h1+h2+1/2TT2 and pAZIL-S-h1+h2+TT2 (Figure 1C and Table 1) were constructed as described above using the appropriate primers (SacI_before_prom-Fw/before_the_loop-hyp2-Rev, SacI_before_prom-Fw/middle_the_loop_hyp2-Rev and SacI_before_prom-Fw/after_the_loop-hyp2-Rev, respectively) (Table 2). MG7284 was transformed with each of the following constructs (pAZIL-S-h1, pAZIL-S-h1+h2-TT2, pAZIL-S-h1+h2+1/2TT2 and pAZIL-S-h1+h2+TT2) and the obtained derivatives (listed in Table 1) were used as bacteriocin producers in the agar well diffusion assay.

For analysis of the P*lliBU* promoter activity, P*lliBU* was fused to *lacZ1* gene in pNZ8150-lacZ1 vector. The P*lliBU* promoter transcriptional fusion was constructed as follows; PCR amplified fragments (primers are listed in Table 2) of promoters P*lliBU* and P*lcnB* (promoter of *lcnB* gene used as a control) were first cloned to pBluescriptT/A giving constructs (pBluescriptT/A-PlliBU and pBluescriptT/A-PlcnB) that were recloned as *Eco*RI-*Bam*HI fragments to pNZ8150-lacZ1 (Table 1). The constructs, designated pNZ8150-PlliBU-lacZ1 and pNZ8150-PlcnB-lacZ1, were used for further experiments. MG7284 was transformed with constructs pNZ8150-PlliBU-lacZ1 and pNZ8150-PlcnB-lacZ1 separately, resulting in the strains MG7284/pNZ8150-PlliBU-lacZ1 and MG7284/pNZ8150-PlcnB-lacZ1. The resulting transformants were screened on solid GM17 medium supplemented with chloramphenicol and X-gal.

### DNA Sequencing and Sequence Analysis

Cloned fragments of all relevant constructs were sequenced by Macrogen Sequencing Service (Macrogen Europe, Amsterdam, Netherlands). The DNA Strider 1.4f7 program was used for sequence analysis and ORF prediction.

### Transformation of *E. coli* and *L. lactis* subsp. *cremoris* MG7284

Standard heat-shock transformation was used for plasmid transformation of *E. coli* competent cells (Hanahan, 1983). *L. lactis* subsp. *cremoris* MG7284 was transformed with plasmid constructs using a method described by Holo and Nes (1989) with modifications. To obtain competent cells, the overnight culture was diluted 100-fold in GM17 medium supplemented with 1% glycine. After growth at 30°C to an optical density at 600 nm OD_600_ = 0.6 to 1.0, the cells were harvested by centrifugation at room temperature at 5,000 × *g*. Following two washes in sterile MilliQ water, the cells were suspended in 1/100 culture volume of sterile MilliQ water. Aliquots (200 μl) of cells were mixed with 5–10 μl of DNA dissolved in 10 mM Tris-hydrochloride (pH 7.5), kept for 5 min at room temperature and then transferred to an electroporation cuvette (2-mm electrode gap) and exposed to a single electrical pulse. The pulse was delivered by a Gene Pulser Eporator (Eppendorf, Hamburg, Germany) set at 25 μF and normally at 2.0 kV. Immediately following the discharge 800 μl of GM17 medium was added to suspensions. After regeneration for 2 h at 30°C appropriate dilutions were spread on selective GM17 plates. Transformants were visible after 1 or 2 days of incubation at 30°C.

For clonal confirmation, pulse-field gel electrophoresis (PFGE) was performed, as described previously by Kojic et al. (2006).

### Bacteriocin Activity Assay

For detection of bacteriocin activity, an agar-well diffusion assay was performed as described previously by Kojic et al. (1991). For testing the level of sensitivity to lactolisterin BU spot on the lawn assay was used; double serial dilutions of purified lactolisterin BU were spotted (5 μl) on the surface of the top agar inoculated with each indicator strain. After 24 h of incubation at 30°C plates were examined for the presence of inhibition zones. A clear zone of inhibition was taken as evidence of bacteriocin production. For monitoring of time dependent production of bacteriocin by different constructs all cultures were started with 1% inoculum from overnight culture and activity of cell free supernatant was tested after different time intervals.

Antimicrobial activity of cytoplasmic fraction was tested in the following way: cells from 50 ml of overnight cultures of bacteriocin producers (BGBU1-4 and MG7284/pAZIL-SB), MG7284/pAZIL-SS and MG7284/pAZIL as negative control were harvested by centrifugation (10 min 5,000 × *g*), washed with 10 ml of PP buffer (0.5 M sucrose, 40 mM NH_4_-acetate, 10 mM Mg-acetate, pH 7), treated with lysozyme (4 mg/ml in PP buffer) 30 min at 37°C to obtain protoplasts. Protoplasts were gently washed with PP buffer and diluted to obtain equal number of cells. 1 ml of each sample was sonicated on ice for different time period (20, 40, and 60 s) with intervals of 10 s per every 20 s of treatment, to disrupt membrane. Cell debris was removed by centrifugation (5 min 20,000 × *g*) and supernatant containing cytoplasmic proteins was used in bacteriocin activity assay. Also cell debris was resuspended in PP buffer and used as negative control in bacteriocin activity assay.

The bacteriocin activity assay was performed at least in two independent repetitions.

### Enzymatic Activity: β-Galactosidase Assay

β-Galactosidase activity was determined essentially as described by Miller (1972) with the modification introduced by Uzelac et al. (2015). The resulting derivative MG7284/pNZ8150-PlliBU-lacZ1 and appropriate controls (MG7284/pNZ8150-PlcnB-lacZ1 and MG7284/pNZ8150-lacZ1) were analyzed with three separate bacterial cultures in two independent experiments.

### RT-PCR

Total mRNA was isolated from *Lactococcus lactis* subsp. *lactis* BGBU1-4 at late logarithmic phase of growth using RNeasy Mini Kit as described by the manufacturer (Qiagen, Hilden, Germany). First strand cDNA synthesis with reverse transcriptase was carried out as previously published by Uzelac et al. (2015). The obtained cDNA was subsequently amplified by PCR using appropriate pairs of primers (Table 2). The size of the obtained PCR products was checked on a 1% agarose gel. Reproducibility of obtained PCR products was confirmed in two independent experiments.

## Results and Discussion

Lactolisterin BU production is plasmid encoded. In a previous study, it was reported that *Lactococcus lactis* subsp. *lactis* BGBU1-4 contains a plasmid, pBU6, carrying the structural lactolisterin BU operon (Lozo et al., 2017). Based on comparisons with known bacteriocin clusters, it was hypothesized that the lactolisterin BU bacteriocin operon was organized into a four gene operon-like structure consisting of (*lliBU*) the structural gene; (*abcT*) an ABC transporter; and *hyp1* and *hyp2* genes to which the exact function has not been attributed.

### Comparative Analysis of Aureocin A53-Like Clusters

**FIGURE 2:**
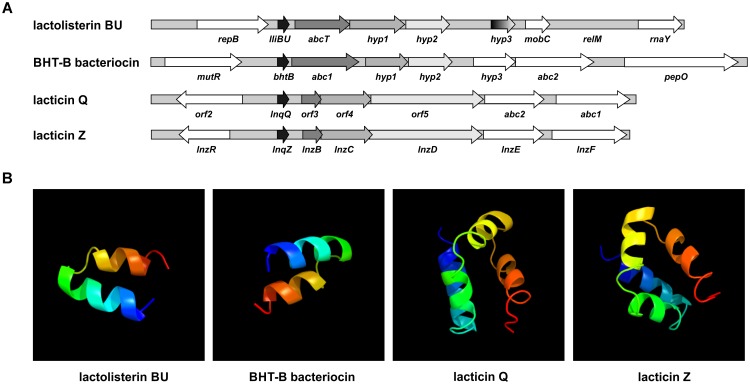
Comparative analysis of aureocin A53-like clusters. **(A)** Schematic presentation of different aureocin A53-like bacteriocins gene clusters. **(B)** Predicted 3D peptide structures for different aureocin A53-like bacteriocins using Phyre2 (http://www.sbg.bio.ic.ac.uk/phyre2).

Since lactolisterin BU showed the highest identity with bacteriocins belonging to aureocin A53-like group (Lozo et al., 2017), the possibility of a common origin was considered. To determine the similarity of the organization of gene clusters among aureocin A53-bacteriocins, lactolisterin BU operon was compared with the three most similar aureocin A53-like bacteriocin clusters: BHT-B bacteriocin from *Streptococcus ratti* (GenBank: DQ145753.1), lacticin Z (GenBank: AB740019.1) and lacticin Q (GenBank: AB712393.1) from *Lactococcus lactis* QU 5. It was found that the lactolisterin BU operon has a unique organization that is distinct from the other aureocin A53-like clusters (Figure 2). Bearing in mind that the aureocin A53-like bacteriocin clusters do not show similarity, it can be assumed that the cluster does not have a common origin. Thus, comparative analysis with known aureocin A53-like clusters, did not help with defining/predicting the exact function of hypothetical genes of the lactolisterin BU cluster.

#### *In silico* Analysis of Genes in Lactolisterin BU Cluster

Downstream of the lactolisterin BU (*lliBU*) structural gene are located an *abcT* gene encoding an ABC transporter ATP-binding protein (domain architecture ID 11438412) and three undefined *hyp1*, *hyp2*, and *hyp3* genes, upstream of the *mobC* gene, predicted to be involved in plasmid mobilization. *In silico* analyses of these three hypothetical genes revealed that *hyp1* is predicted to encode a putative sugar ABC transporter permease of 212 amino acid residues, but that *hyp2* and *hyp3* encode proteins of 169 and 92 amino acids that share low level similarity with uncharacterized proteins from LAB.

TMpred program prediction^1^ analysis of those three proteins revealed the presence of transmembrane helices (Hyp1 = 6; Hyp2 = 5 and Hyp3 = 2), indicating a cytoplasmic membrane location for these proteins. In addition, it was found that all three proteins have high pI values (Hyp1 = 10.52; Hyp2 = 9.77; and Hyp3 = 11.05) and are rich in the amino acid leucine (20.5; 11.8; and 15.5%, respectively).

In the lactolisterin BU cluster, two genes were predicted to encode possible transporter/enzymes involved in sugar catabolism, indicating that the lactolisterin BU peptide determinant was somehow introduced upstream of a sugar-related cluster and uses these proteins for its own transport.

The absence or existence of small intergenic regions between the genes of lactolisterin BU operon indicated the synthesis of a single polycistronic RNA (the intergenic region between the *lliBU* and *abcT* genes is 64 nucleotides; the *abcT* and *hyp1* genes are overlapped within 8 nucleotides; the intergenic region between the *hyp1* and *hyp2* genes is only 3 nucleotides).

*In silico* analysis of the upstream region of lactolisterin BU operon using the program Bacterial Promoter Prediction^2^ revealed the presence of five putative promoter structures of which (TGAATATTTGCAATTTTTATATTAAATT) had the highest score (1) and, thus the greatest likelihood of being active.

Analysis to detect the presence of transcriptional terminators (TT) using^3^ revealed the presence of two apparent transcriptional terminators in the lactolisterin BU operon: TT1 within the intergenic region of the *lliBU* and *abcT* genes (GGTTAAAATATAATCTCGGAATGCATTAATATGCATCCCGAGATTATATTTTAACC) and second a TT2 after the *hyp2* gene at the end of operon (TTTCCGCCAAAAAAAGTAAGCTATTCGCTTACTTTTTTTGGCGGAAA). The presence of two transcription terminators in the lactolisterin BU operon also pointed to the possibility that the first TT1 was introduced together with the bacteriocin structural gene during insertion into the sugar operon. We hypothesize that it was later modified in order to provide a precise regulation of expression by transcriptional attenuation.

Upstream of the *lliBU*, *abcT, hyp1* and *hyp2* at a variable distance from the ATG translation start codon non-perfect ribosomal binding sequences (RBSs) were detected (Table 3). These RBS could be involved in translational regulation of expression of each gene in the cluster.

**Table 3:** Position and sequence of RBS upstream of genes in lactolisterin BU operon.

Gene	Distance from ATG	Sequence	Consensus
*lliBU*	11	TGGAGG	AGGAGG
*abcT*	6	AGGAGAG	
*hyp1*	8	TGGAGCG	
*hyp2*	8	ACGAAGG	

### Transcriptional Analysis of the Lactolisterin BU Operon

**FIGURE 3:**
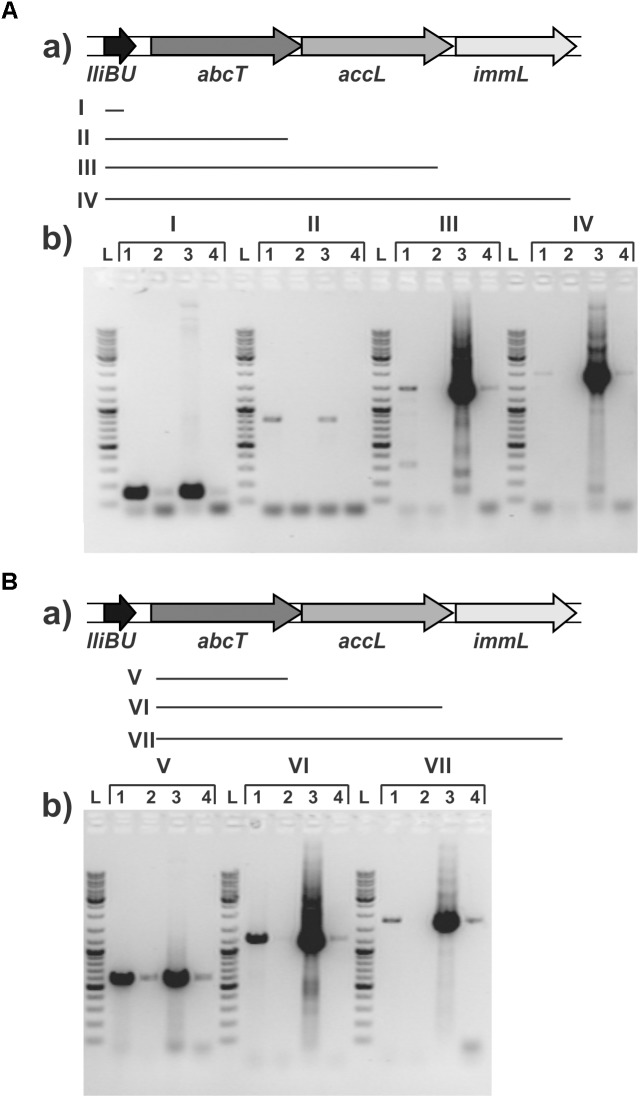
Transcriptional analysis of the lactolisterin BU operon. Amplicons obtained after RT-PCR using **(A)** lliBU-Fw and (I) lliBU-Rev; (II) abcT-Rev; (III) accBU-Rev; (IV) immBU-Rev; **(B)** abcT-Fw and (V) abcT-Rev; (VI) accBU-Rev; (VII) immBU-Rev primers; **(a)** schematic presentation of primer positions and length of PCR products in lactolisterin BU operon; **(b)** electrophoresis of obtained PCR products on 1% agarose gel; L – GeneRuler DNA Ladder Mix 0.1–10 kb (Thermo Fisher Scientific); 1 – cDNA of BGBU1-4; 2 – RT-PCR negative control (without reverse transcriptase); 3 – total DNA of BGBU1-4; 4 – PCR negative control (without DNA of BGBU1-4).

In order to test if all genes (*lliBU*, *abcT*, *hyp1*, and *hyp2*) for lactolisterin BU activity form an operon (transcribed as one polycistronic mRNA), their expression was investigated by RT-PCR using one forward primer from the *lliBU* gene and different reverse primers positioned in the *lliBU*, *abcT*, *hyp1* or *hyp2* genes. Due to the presence of a strong transcriptional terminator immediately after the *lliBU* gene, an additional forward primer from the *abcT* gene was designed and used for transcriptional analysis. The results of RT-PCR analysis unambiguously showed that all genes of the lactolisterin BU cluster were transcribed as a single polycistronic mRNA (Figure 3). This was in line with the previously characterized bacteriocin cluster for enterocin AS-48 that is expressed by two polycistronic mRNA in coordinate expression (Diaz et al., 2003).

Considering that production of lactolisterin BU in the strain *L. lactis* subsp. *lactis* BGBU1-4 is balanced at low level since higher production of bacteriocin cause the death of the producer (Lozo et al., 2017), we wanted to determine the level of expression at the transcriptional level through the construction of the promoter P*lliBU-lacZ* fusion and the analysis of the activity. The promoter P*lcnB* of the lactococcin B gene was used as a positive control. Results of β-galactosidase activity of transcriptional fusions revealed that promoter P*lliBU* had activity comparable to promoter P*lcnB* (the difference in activity between the MG7284/pNZ8150-PlcnB-lacZ1 and MG7284/pNZ8150-PlliBU-lacZ1 did not have statistical significance; 183 ± 21: 169 ± 26 MU, respectively). This result indicates that regulation of expression was more likely to be at the translational level.

#### Cloning of Lactolisterin BU Operon in the pAZIL Vector

**FIGURE 4:**
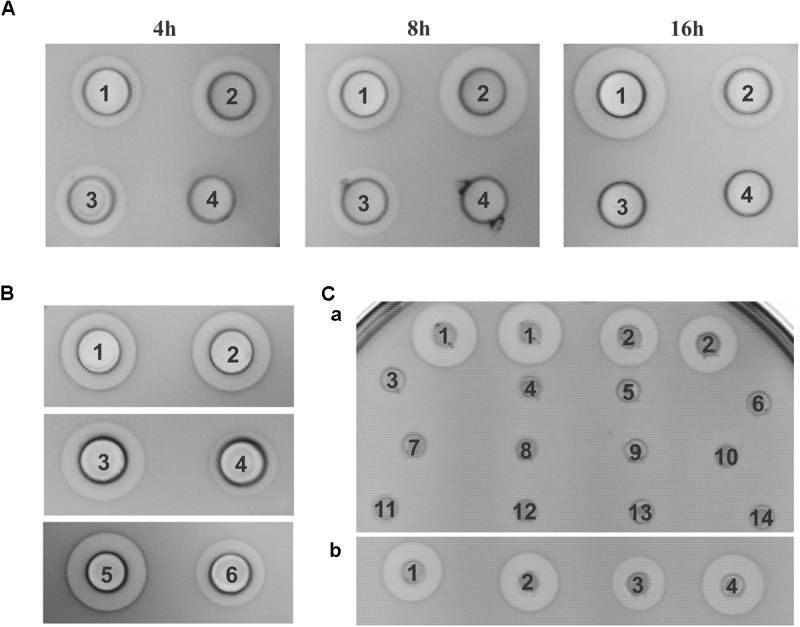
Antimicrobial activity of constructed derivatives on *Lactococcus lactis* subsp. *cremoris* MG7284. **(A)** Time dependent antimicrobial activity of MG7284/pAZIL 1 – SP, 2 –SB, 3 – BGBU1-4, 4 – MG7284. **(B)** Dependence of cluster orientation cloned into pAZIL vector on antimicrobial activity: MG7284/pAZIL 1 – pBU6P-1, 2 – pBU6P-5, 3 – pBU6B-2, 4 – pBU6B-4, 5 – SP, 6 – SB. **(C)** Bacteriocin activity of lactolisterin BU cluster expressed *in trans* **(a)** MG7284/pAZIL 1 – SP, 2 – SB, MG7284/3 – pNZ8150-h1, 4 – pAZIL-SS+pNZ8150-h1, 5 – pNZ8150-h2, 6 – pAZIL-SS+pNZ8150-h2, 7 – pNZ8150-h1+h2, 8 – pAZIL-SS+pNZ8150-h1+h2, 9 – pNZ8150-EEB, 10 – pAZIL-SS+pNZ8150-EEB, 11 – pNZ8150-h3, 12 – pAZIL-SS+pNZ8150-h3, 13 – pAZIL-SS, 14 – MG7284; **(b)** MG7284/pAZIL 1 – SP, 2 – SP+pNZ8150-h3, 3 – SB, 4 – SB+pNZ8150-h3.

Lactolisterin BU is encoded by a gene cluster located on the smallest plasmid in strain BGBU1-4, pBU6 of 6.2 kb (Figure 1A). To characterize a minimal bacteriocin cluster, different fragments of pBU6 plasmid were cloned in a pAZIL shuttle vector (Figure 1B and Table 1). The smallest fragment providing bacteriocin activity in heterologous strain MG7284 was the 2,552 bp *Sac*I-*Bgl*II fragment containing the *lliBU*, *abcT*, *hyp1* and *hyp2* genes (Figures 1B, 4). It was interesting that the construct pAZIL-SB (pAZIL vector containing *Sac*I-*Bgl*II fragment) exhibited stronger antimicrobial activity than the wild type strain and transformants of MG7284 with pBU6 plasmid. Construct pAZIL-SP (pAZIL vector containing the *Sac*I-*Pvu*II fragment) showed even better antimicrobial activity (higher zone of inhibition; Figure 4) than a transformant with pAZIL-SB construct indicating the influence of other genes/structures on bacteriocin activity. Plasmid construct pAZIL-SS (pAZIL containing *Sac*I–*Sca*I fragment of 1,284 bp) carrying only the *lliBU* and *abcT* genes did not provide antimicrobial activity, indicating that additional gene(s) were needed for bacteriocin production. In order to prove this conclusion we performed bacteriocin activity assay with cytoplasmic fraction of different strains: bacteriocin producers (BGBU1-4 and MG7284/pAZIL-SB), MG7284/pAZIL-SS and MG7284/pAZIL as negative control. Very low bacteriocin activity (1 mm of inhibition zone) was obtained only with strains that already produce bacteriocin in supernatant (BGBU1-4 and MG7284/pAZIL-SB), which was originated from residual bacteriocin molecules that were not exported (no bacteriocin activity was obtained with cell debris) strongly indicated that no bacteriocin synthesis occurs inside of the cells carrying pAZIL-SS construct, although the structural bacteriocin gene is present and an active promoter. According to both *in silico* analysis (all four genes are transcribed as polycistronic mRNA) and subcloning results, it can be concluded that the operon is located on the *Sac*I-*Bgl*II fragment and consists of four structural genes: *lliBU*, *abcT*, *hyp1* and *hyp2*. In addition, the time-dependent production of bacteriocin, that was noted in the parental BGBU1-4 strain (Lozo et al., 2017), was also tested in derivatives MG7284/pAZIL-SP and MG7284/pAZIL-SB. It was observed that derivative MG7284/pAZIL-SB retained time-dependent production of bacteriocin, like the parental strain (although bacteriocin production was higher), while MG7284/pAZIL-SP lost time-dependent production of the bacteriocin (Figure 4A). In addition to the role of specific genes, the influence of the orientation of the fragment in the vector on bacteriocin expression was observed (Figure 4B) indicating that some (promoter or other) sequences from the vector could boost the expression. Thus, it was noticed that depending on the orientation of the same fragment (*Bgl*II linearized plasmid pBU6), expression of bacteriocin could drastically differ.

#### The *hyp1* Gene Encodes an Accessory Protein

In order to determine the role of the *hyp1* gene, we made additional constructs (Figures 1B–E and Table 1). The most important construct for determining the function of the *hyp1* gene was pAZIL-S-h1 (containing a cloned PCR fragment carrying P*lliBU*, *lliBU*, *abcT*, and *hyp1* genes). Transformants were successfully obtained in *E. coli*, but no transformants were obtained in MG7284 after three independent attempts. In control transformations with the same competent cells, but with other constructs (pAZIL-SS, pAZIL-SB, and pAZIL-SP), we obtained between 6 × 10^4^ and 2 × 10^5^/μg of plasmid DNA. By combining the obtained results with respect to the activity of subclones, it can be concluded that the *hyp1* gene encoded for the accessory protein and was designed as the *accL* gene (Figure 1F). The combined evidence is as follows (*i*) the pAZIL-SS construct did not provide antimicrobial activity because it lacked a key protein for delivery of bacteriocin, an accessory protein, (*ii*) construct pAZIL-S-h1 contained all genes necessary for synthesis and transport of bacteriocin and provided transformants to produce active bacteriocin that killed themselves due to the absence of the immunity protein. These results explain why in the first case transformants that lacked bacteriocin production capacity were generated and in the second, we were not able to get them at all. The location of an accessory protein downstream from the gene for ABC transporter was previously confirmed for the bacteriocin BacSJ (Kojic et al., 2010) and many other class II bacteriocins (Nes et al., 1996) and that it is essential for export of bacteriocins in addition to the ABC transporter. In lactolisterin BU and BHT-B operons accessory protein resembles permease enzymes which can contribute to streamlining its role in bacteriocin transport and interaction with ABC transporter.

#### The *hyp2* Gene Is Responsible for Immunity to Lactolisterin BU

A construct containing the *hyp2* gene was the only one that had the ability to synthesize and increase resistance to lactolisterin BU. In addition, for construct pAZIL-S-h1, which contains the operon without the *hyp2* gene, it was not possible to obtain any transformants due to the apparent absence of immunity.

**FIGURE 5:**
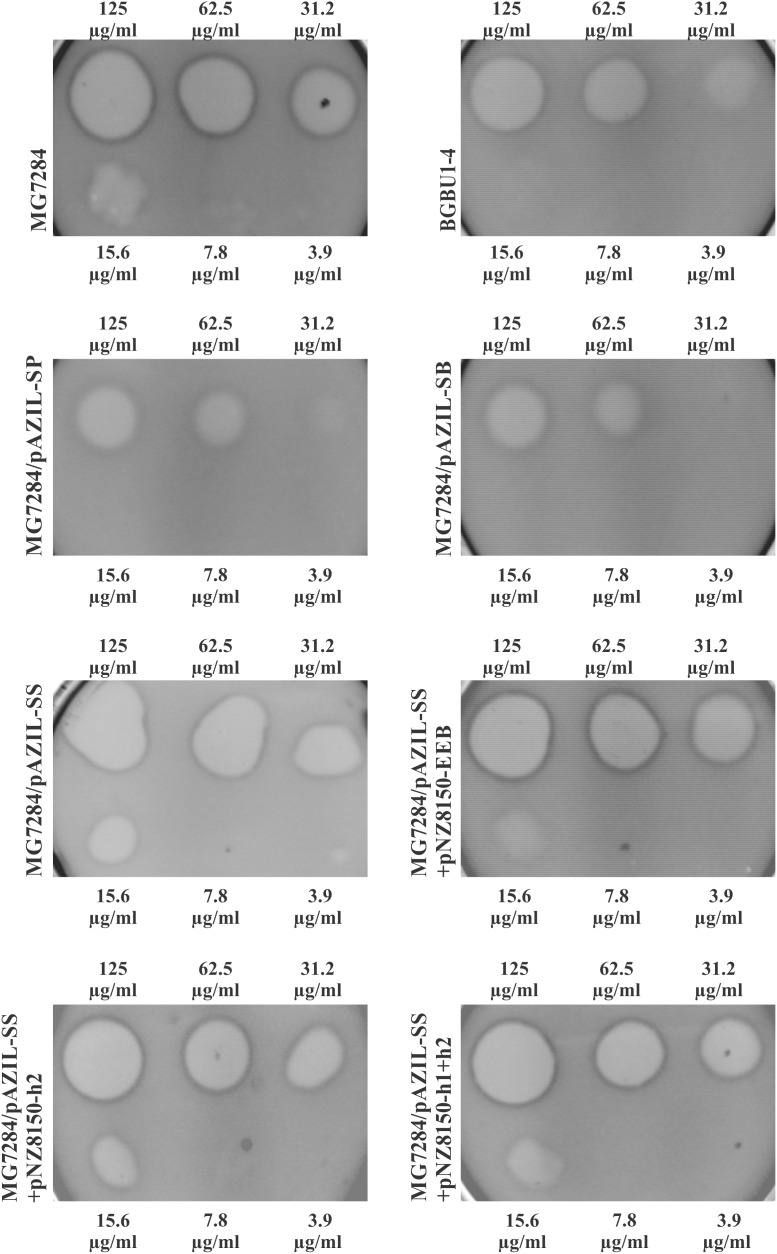
Sensitivity of representative strains and derivatives to different concentrations of purified lactolisterin BU in spot on the lawn bacteriocin assay.

To determine if the *hyp2* gene product (alone) can provide immunity to lactolisterin BU, this gene was amplified and cloned under the control of the P*lliBU* promoter, transferred to pNZ8150 vector and expressed in MG7284. The transformants carrying this construct were exposed to different concentrations (500, 250, 125, 62.5, 31.2, 15.6, 7.8, and 3.9 μg/ml) of purified lactolisterin BU and it was found that all of them were as sensitive as the sensitive strain MG7284 (Table 4). To further investigate the possible role of the *hyp2* gene product in providing immunity to lactolisterin BU all constructs were exposed to different concentrations (500, 250, 125, 62.5, 31.2, 15.6, 7.8, and 3.9 μg/ml) of purified lactolisterin BU in a spot on the lawn bacteriocin assay. It was observed that constructs containing a functional *hyp2* gene showed approximately four times higher resistance to bacteriocin then “sensitive strains”- i.e., the same or even higher than the parental strain-producer of lactolisterin BU (Figure 5 and Table 4). Since the function of the *hyp2* gene was determined to be to provide immunity to lactolisterin BU, the gene was renamed *immL* (Figure 1F). Notably, a limited level of protection against its own synthesized antimicrobial molecule was noted for aureocin A53-like bacteriocins previously (Iwatani et al., 2007). Most likely it is the limiting factor for bacteriocin production, which must be well balanced in order not to kill itself. It could be assumed that low level of immunity to lactolisterin BU is result of balanced translational regulation or still ongoing transition of sugar to bacteriocin operon.

**Table 4:** Sensitivity of all analyzed derivatives to different concentrations of purified lactolisterin BU in spot on the lawn bacteriocin assay.

Name of derivative	Concentration of purified lactolisterin BU (μg/ml)					
	125	62.5	31.2	15.6	7.8	3.9
BGBU1-4	+	+	+	−	−	−
BGMN1-596	+	+	+	+	−	−
MG7284	+	+	+	+	−	−
MG7284/pBU6	+	+	+	−	−	−
MG7284/pAZIL-SP	+	+	+/−	−	−	−
MG7284/pAZIL-SB	+	+	+/−	−	−	−
MG7284/pAZIL-SS	+	+	+	+	+/−	−
MG7284/pAZIL-pBU6P-1	+	+	+	−	−	−
MG7284/pAZIL-pBU6P-5	+	+	+	−	−	−
MG7284/pAZIL-pBU6B-2	+	+	+	−	−	−
MG7284/pAZIL-pBU6B-4	+	+	+	−	−	−
MG7284/pNZ8150-h1	+	+	+	+	−	−
MG7284/pAZIL-SS+pNZ8150-h1	+	+	+	+	−	−
MG7284/pNZ8150-h2	+	+	+	+	−	−
MG7284/pAZIL-SS+pNZ8150-h2	+	+	+	+	−	−
MG7284/pNZ8150-h1+h2	+	+	+	+	−	−
MG7284/pAZIL-SS+pNZ8150-h1+h2	+	+	+	+	−	−
MG7284/pAZIL-SB+pNZ8150-h3	+	+	+/−	−	−	−
MG7284/pAZIL-SB+pNZ8150-h3+mobC+relM+rnaY	+	+	+/−	−	−	−
MG7284/pNZ8150-EEB	+	+	+	+	−	−
MG7284/pAZIL-SS+pNZ8150-EEB	+	+	+	+	−	−
MG7284/pAZIL-S-h1+h2-TT2	+	+	+/−	−	−	−
MG7284/pAZIL-S-h1+h2+1/2TT2	+	+	+/−	−	−	−
MG7284/pAZIL-S-h1+h2+TT2	+	+	+/−	−	−	−

### The Transcription Terminator TT2 Did Not Influence the Expression of Lactolisterin BU in Lactococci

It has been documented that transcription terminators can be involved in regulation of gene expression by transcriptional attenuation or impacting on the stability of mRNA originating from bacteriocin operons (Uzelac et al., 2015). Different constructs were made to determine the possible involvement of TT2 in the expression of lactolisterin BU (Figure 1C). It was found that constructs without TT2 expressed the same level of bacteriocin (the same size of the zone of inhibition), indicating that TT2 was not involved in additional regulation of lactolisterin BU expression.

### Product of the *hyp3* Gene Modulates Expression of Lactolisterin BU

The possible involvement of the *hyp3* gene in expression of lactolisterin BU was also analyzed (Figure 1E). During subcloning of the pBU6 plasmid, it was noticed that construct pAZIL-SP provided a larger zone of inhibition than pAZIL-SB (that contains the perceived complete lactolisterin gene cluster), despite the growth of bacteriocin producer MG7284/pAZIL-SP being lower. In addition, when the *hyp3* gene was subcloned into the pNZ8150 vector and co-expressed with pAZIL-SB in MG7284, it provided the same level of lactolisterin BU production as MG7284/pAZIL-SP (Figure 4Cb). The *hyp3* gene did not belong to lactolisterin BU operon, but may somehow modulate expression or transport of lactolisterin BU, possibly due its membrane location.

### The Lactolisterin BU Operon Expressed *in trans* Does Not Provide Bacteriocin Activity

Subcloning experiments with the pBU6 plasmid showed that the lactolisterin BU gene cluster consists of four genes (*lliBU*, *abcT*, *accL* and *immL*) clustered as one transcriptional unit. To test the possibility of *in trans* expression by different combinations of lactolisterin BU genes, the following experiments were completed: the lactolisterin BU operon was split and cloned into two compatible vectors with different selection markers (pAZIL and pNZ8150) carrying different parts (genes) of the cluster (*lliBU* + *abcT*, *hyp1* and *hyp2*; *lliBU* and *abcT* + *hyp1* and *hyp2*; *lliBU* and *abcT* + *hyp1*; *lliBU* and *abcT* + *hyp2*) (Figures 1E, 4Ca). Transcription of the first part of the operon was driven by the natural promoter P*lliBU*, while the second part was also constructed to be transcribed under the same promoter. Results of bacteriocin testing showed that combinations of *in trans* co-expressed lactolisterin BU genes were not able to restore bacteriocin activity, indicating an obligate *in cis* gene constellation. This indicates that bacteriocin expression is controlled by a translational coupling mechanism, providing an optimal ratio of proteins within the operon to obtain enough bacteriocin molecules for combating competitors, but not so much as to kill itself due to limited immunity.

The results of this study highlight that sophisticated mechanisms of regulation were involved in expression of genes within the lactolisterin BU cluster, which require further study. Elucidation of regulatory mechanisms involved in the expression of lactolisterin BU could help in making constructs for the overexpression of bacteriocins and/or other proteins of interest.

## Conclusion

The lactolisterin BU cluster consists of four genes (*lliBU*, *abcT*, *accL*, and *immL*) clustered as one transcriptional unit under the P*lliBU* promoter. Gene product functions were determined to be: *lliBU* – the bacteriocin structural gene, *abcT –* the ABC transporter, *accL* – the transporter accessory protein and *immL* – an immunity protein which provides limited protection. P*lliBU* promoter activity was determined to be as strong as that of P*lcnB*, indicating that expression is predominantly regulated at the translational level. The absence of activity when the gene cluster was divided and expressed *in trans* indicates that bacteriocin expression is controlled to provide an optimum ratio of protein expression levels within the operon. The operon functions *in cis* constellation of genes ensuring the optimal amount of each product for the cell most probably by a translational coupling mechanism. In addition, it was found that the presence of the hypothetical gene/protein 3, located downstream of the lactolisterin BU operon (possesses its own promoter) has a positive effect on the bacteriocin expression.

## Author Contributions

MaM and MK conceived, designed, and coordinated this study, interpreted all of results and contributed to the preparation of the figures, and wrote this paper. JL, NM, MiM, and BJ provided experimental assistance and contributed to the preparation of the figures and manuscript. PO performed experiments of bacteriocin purification. PO and PC provided technical assistance and contributed to the preparation of this paper. All authors reviewed the results and approved the final version of the manuscript.

## Conflict of Interest Statement

The authors declare that the research was conducted in the absence of any commercial or financial relationships that could be construed as a potential conflict of interest.

## Footnotes

1 https://embnet.vital-it.ch/software/TMPRED_form.html

2 http://www.bacpp.bioinfoucs.com/ferramenta

3 http://rna.igmors.u-psud.fr/toolbox/arnold/
